# COVID 19: challenges for virologists in the food industry

**DOI:** 10.1111/1751-7915.13638

**Published:** 2020-07-22

**Authors:** Sophie Zuber, Harald Brüssow

**Affiliations:** ^1^ Institute of Food Safety and Analytical Science Nestlé Research Lausanne 26 1000 Switzerland; ^2^ Department of Biosystems Laboratory of Gene Technology KU Leuven Leuven Belgium

## Abstract

The COVID‐19 pandemic is not only a challenge for public health and hospitals, but affects many aspects of our societies. This Lilliput minireview deals with problems that the pandemic causes for the food industry, addressing the presence and persistence of SARS‐CoV‐2 in the food environment, methods of virus inactivation and the protection of the food worker and the consumer. So far food has not been implicated in the transmission of the infection, but social disruptions caused by the pandemic could cause problems with food security.

## Introduction

The epidemiology of the ongoing COVID‐19 pandemic clearly indicates that the main route of transmission of SARS‐CoV‐2 is human‐to‐human by close contact through respiratory droplets (Chu *et al*., 2020). Uncertainties around additional modes of transmission such as the importance of fomite transmission and the possibility of aerosol transmission have raised questions in many business sectors, but have put a particular strain on the different players in the food supply chain, as many questions remain around the persistence and inactivation of SARS‐CoV. This review aims at giving an overview of current and future challenges and knowledge gaps on the virus presence in non‐medical environments, including the presence of SARS‐CoV in sewage, the role of animals in the ongoing pandemic, persistence of the virus on surfaces, virus inactivation technologies and how best to protect food workers and consumers.

## Presence of SARS‐CoV‐2 in the environment

### Faeces

The presence of SARS‐CoV‐2 in faecal material has been demonstrated in COVID‐19 patients. Patients from Singapore, who showed a relatively mild disease, demonstrated a median duration of virus shedding of 12 days and half of the patients excreted virus into the stool, but not urine (Young *et al*., 2020). Faeces have been shown to contain SARS‐CoV‐2 three days after infection, well before symptoms appeared (Mallapaty, 2020b). An important proportion of patients show gastrointestinal symptoms (Jin *et al*., 2020), but it is still unclear whether SARS‐CoV‐2 viruses present in stool are mostly defective particles containing RNA or intact and infectious virions. Isolation of infectious virus from stool was described in one study (Wang *et al*., 2020c), but not in another (Wölfel *et al*., 2020), and no cases of faecal–oral transmission of COVID‐19 have been reported.

### Sewage water

In The Netherlands, untreated sewage samples of 7 cities and Schiphol airport were tested using RT‐qPCR (Medema *et al*., 2020). No SARS‐CoV‐2 RNA was detected in samples of February 6, three weeks before the first case was reported in The Netherlands on February 27. On March 15/16, viral RNA fragments were detected in sewage of six sites. The detection of the virus in sewage has been shown in different countries worldwide (Ahmed *et al*., 2020; Randazzo *et al*., 2020; Wu *et al*., 2020) and indicates that sewage surveillance can be used as a sensitive tool to monitor the circulation of the virus in the population and help authorities to coordinate the exit strategy to gradually lift its coronavirus lockdown.

The presence of viral RNA in sewage water not only suggests the need to better understand wastewater as potential sources of epidemiological data but also to better evaluate human health risks. A comprehensive review listed research gaps and future research needs in this field (Kitajima *et al*., 2020). One of the major challenges in SARS‐CoV‐2 detection/quantification in wastewater samples is the lack of an optimized and standardized protocol. First detection of SARS‐CoV‐2 RNA in untreated wastewater in Spain validated an adsorption–precipitation concentration method and showed that 11% secondary treated water samples tested positive for at least one SARS‐CoV‐2 RT‐qPCR target with average RNA titres of 1 × 10^5^–3 × 10^5^ RNA genome copies per litre. Importantly, none of the tertiary effluent samples (*n* = 12) tested positive for the presence of SARS‐CoV‐2 RNA (Randazzo *et al*., 2020). This is in contrast with another study in which 6 out of 8 samples from treated wastewater scored positive by RT‐qPCR (Wurtzer *et al*., 2020). It will be important to determine whether isolation of infectious SARS‐CoV‐2 from wastewater is possible, as the SARS epidemic from 2003 had epidemiological links to water and wastewater in a multi‐storey building in Hong Kong involving over 300 people who were all linked to a faulty sewage system (Peiris *et al*., 2003; Gundy *et al*., 2008).

### Sludge

A preprint report describes the quantification of SARS‐CoV‐2 RNA in primary and waste activated sludge samples taken from major municipal wastewater treatment plants in Istanbul. All samples tested positive with titres of SARS‐CoV‐2 ranging between 1 × 10^4^ to 4 × 10^4^ RNA genome copies per litre (Alpaslan Kocamemi *et al*., 2020). It is currently not possible to precisely define an acceptable level of contamination for untreated sludge, or to specify a storage period beyond which the virus is inactivated. The French Agency for Food, Environmental and Occupational Health and Safety (ANSES, 2020b) therefore recommended that sewage sludge produced during the epidemic episode should not be applied to fields without first being heat‐treated. Conducting a quantitative microbial risk assessment (QMRA) for SARS‐CoV‐2 exposure pathways, e.g. through wastewater or sludge, is currently difficult, mainly because the minimal infectious dose for humans is still unknown (Kitajima *et al*., 2020).

## The role of animals in the pandemic

### Wild animals – animal reservoir

The animal reservoir and intermediate species for SARS‐CoV‐2 transmission to humans is still uncertain. The closest bat coronavirus isolate shared a common ancestor with SARS‐CoV‐2 fifty years ago, pangolin coronavirus is even more distantly related (Mallapaty, 2020a; Zhang *et al*., 2020; Zhou *et al*., 2020). Six out of 18 Malayan pangolins intercepted from smugglers yielded coronaviruses that shared 85–92% nucleotide sequence identity with the Wuhan SARS‐CoV‐2 isolate. However, over the receptor‐binding domain (RBD) these pangolin viruses were more closely related to the human isolates than bat coronaviruses, suggesting pangolins as a possible intermediate host involved in the outset of the pandemic (Lam *et al*., 2020). To prevent viruses spilling over from animals to humans, for example in wet food markets, wildlife sources need to be monitored and Volpato and coauthors suggest there is an urgent need for a ban on illegal pangolin but also other wild animal trade to prevent further viral spillovers (Volpato *et al*., 2020; Xiao *et al*., 2020). The WHO has issued recommendations to reduce risk of transmission of emerging pathogens from animals to humans in live animal markets or animal product markets (WHO, 2020b).

### Animal models

Ferrets are a popular model for respiratory viral infections of humans including influenza and SARS‐CoV and crucial for the development of vaccines. Ferrets developed fever after intranasal inoculation with a virus from a COVID‐19 patient, showed moderate titres of virus in the nose and lower titres in lung and intestine, and displayed pathology in the lung tissue. Ferrets recovered after 2 weeks and seroconverted with neutralizing antibodies. Infected ferrets transmitted the infection efficiently in the pre‐symptomatic stage to naïve ferrets by close contact, but also, albeit less efficiently to ferrets in separate cages by the droplet route (Kim *et al*., 2020). This was also observed for hamsters. When naïve hamsters were placed into a soiled cage without the infected hamster, transmission by contaminated fomites was observed, but was less efficient (only one out of three naïve hamsters was infected; Sia *et al*., 2020).

### Pet animals

Early in the pandemic, the veterinary services of Hong Kong reported to the World Organisation for Animal Health (OIE, Office International des Epizooties) the RT‐qPCR detection of SARS‐CoV‐2 in nasal and oral specimens of two asymptomatic dogs owned by COVID‐19 patients and a few weeks later the Belgian veterinary services reported a symptomatic cat that tested positive by sequencing for SARS‐CoV‐2 in stool and vomit and belonged to a COVID‐19 owner (OIE, 2020). Chinese scientists inoculated a number of animal species with both an environmental virus isolate from the wet food market in Wuhan and an isolate from an early patient from Wuhan (Shi *et al*., 2020). Ferrets could be infected and developed an upper respiratory tract infection with fever. Outbred domestic cats could also be infected and developed respiratory tract symptoms and specific antibodies. Cats could transmit infection via the airborne (droplet) route to other cats. Viral RNA, but not infectious virus, was detected in the intestine of infected cats. Young cats were shown to be more susceptible to infection than adult cats. Dogs showed after infection viral RNA in the intestine, but not in the lung. No infectious virus was found in the gut of dogs, indicating low susceptibility of dogs for this virus and it is not known whether the doses of SARS‐CoV‐2 used to initiate the experimental infections of the animals tested would be achieved in a natural setting. The OIE recommends that people who are sick with COVID‐19 limit contact with companion and other animals, emphasizing that when handling and caring for animals, basic hygiene measures should always be implemented. This includes hand washing before and after being around or handling animals, their food, or supplies, as well as avoiding kissing, and licking animals or sharing food (OIE, 2020).

### Farm animals

Pigs, chicken and ducks could not be infected with SARS‐CoV‐2 (Shi *et al*., 2020). OIE and CDC communicated that rare cases of human‐to‐animal SARS‐CoV‐2 transmission have been reported, including a tiger in a New York zoo which contracted the infection from an infected human (OIE, 2020). A preprint article reports infections on two mink farms with SARS‐CoV‐2 in The Netherlands (Oreshkova *et al*., 2020). In both farms, COVID‐19‐like symptoms were present in individuals working on the farms before clinical signs in mink were seen. Transmission between minks is suspected in this farm setting through fomites (e.g. by feed or bedding material provided by humans), by infectious droplets generated by the infected animals, or by (faecally) contaminated dust from the bedding. A survey of 4000 samples from dogs, cats and horses from places where community transmission of COVID‐19 occurred in humans were all negative, suggesting that the virus is not widely circulating in pet and farm animals (Sit *et al*., 2020). There is currently no evidence that companion and farm animals play a significant role in the transmission of the epidemic (ANSES, 2020a; OIE, 2020).

## Persistence of SARS‐CoV‐2

### Water

SARS‐CoV‐2 in virus transport medium (optimal conditions for virus survival) is highly stable at 4°C (< 0.7 log_10_ reduction on day 14; Chin *et al*., 2020). This is similar to experiments carried out with SARS‐CoV which demonstrated that the virus could survive for up to 14 days at 4°C in hospital wastewater, domestic sewage and dechlorinated tap water, but only persists for 2 days at 20°C (Wang *et al*., 2020b). Similarly, a human coronavirus causing common cold (HCoV 229E) remained infectious for 10–100 days in tap water, yet for only 2–4 days in wastewater as determined by TCID_50_ (tissue culture infectious dose 50% technique; Gundy *et al*., 2008). Shellfish is able to bio‐accumulate viral particles from a large volume of water and is often consumed uncooked (Bosch *et al*., 2018). Thus, surveillance of SARS‐CoV‐2 in seawater and shellfish should be done to estimate a potential risk of this food category for food handlers and consumers through fomite transmission when touching and preparing it. Similarly, surveillance of SARS‐CoV‐2 in agricultural water (e.g. canal, stream, or pond water) could help to better understand the level of risk infectious SARS‐CoV‐2 (if present in in these waters) could have for farm workers and produce, as agricultural water may be used for irrigation, pesticide and fertilizer preparation, cleaning of equipment, and hand washing (Uyttendaele *et al*., 2015; Julien‐Javaux *et al*., 2019).

### Surfaces

Virus transmission via fomites might play a role in the ongoing pandemic, but the relative importance between person‐to‐person, droplet and fomite transmission has not been elucidated. Thus, persistence data of SARS‐CoV‐2 on inanimate surfaces are very valuable for future quantitative microbial risk assessment (QMRA) on SARS‐CoV‐2 exposure pathways. The persistence of SARS‐CoV‐2 was shown to be higher on plastic and stainless steel than on copper and cardboard surfaces, and viable virus was detected up to 72 h after application to these surfaces (van Doremalen *et al*., 2020). The estimated median half‐life of SARS‐CoV‐2 was approximately 5.6 h on stainless steel and 6.8 h on plastic. However, the same authors showed that half‐life for SARS‐CoV‐2 in surface nasal mucus and sputum at 21°C/40% relative humidity (RH) was considerably shorter (3.1 h) and the virus was generally more stable at cooler temperatures and lower RH (Matson *et al*., 2020). Another study showed that at room temperature (22°C) with a relative humidity of around 65%, no infectious virus could be recovered from: printing and tissue papers after 3 h, treated wood and cloth on day 2, glass and banknote on day 4, stainless steel and plastic on day 7 (Chin *et al*., 2020). Differences observed between the studies may partly be due to differences in inoculation levels, experimental protocols and environmental variables. More importantly, the study by van Doremalen and coauthors revealed that the stability of SARS‐CoV‐2 and SARS‐CoV‐1 is very similar and indicates that the greater transmissibility observed for the new virus is not due to greater environmental stability compared to SARS‐CoV‐1.

### Air

Virus persistence data in air from natural settings are so far only known from hospital environments. Surface and air samples from an intensive care unit and a general COVID‐19 ward at a hospital in Wuhan, China, were analysed for SARS‐CoV‐2. Virus RNA was widely distributed on floors, computer mice, trash cans and sickbed handrails and was detected in air around 4 m from patients (Guo *et al*., 2020). A laboratory study showed that SARS‐CoV‐2 remained viable in aerosols throughout the duration of the experiment (3 h), with a reduction in infectious titre of 0.8 log_10_ per litre of air (van Doremalen *et al*., 2020). The results indicate that aerosol transmission of SARS‐CoV‐2 is possible, since the virus can remain viable and infectious in aerosols for hours, but transmission by aerosols has not been proven yet and a US study which found viral RNA in air samples could not infect cell cultures (Lednicky *et al*., 2020). Another study showed that despite extensive environmental contamination, SARS‐CoV‐2 was not present in air samples obtained from the rooms of hospitalized patients with COVID‐19 (Rubens *et al*., 2020).

### Food

Studies looking at the stability of coronaviruses in foods are scarce. Middle East respiratory syndrome coronavirus (MERS‐CoV) which was first reported in Saudi Arabia in 2012 survived in dromedary milk at 4°C for 7 h, while it lost infectivity at 22°C within 48 h of storage (van Doremalen *et al*., 2014). A study used a bovine CoV strain as a model to examine the stability and the potential for foodborne transmission of CoV; the virus was generally more stable at cooler temperatures and lower RH (Mullis *et al*., 2012). Viral RNA was detected from the lettuce surface at all time points (up to 30 days). The infectivity of recovered virus varied significantly depending upon the type of liquid used to prepare the virus stock. From day 2 onwards, no infectious virus was recovered from lettuce spiked with virus suspended in a 10% faecal suspension. In contrast, infectious virus was detected for up to 25 days on lettuce spiked with virus suspended in artificial cell culture medium. In another study, HCoV 229E could not be recovered from lettuce after 4 days of storage at 4°C (Yépiz‐Gómez *et al*., 2013). Data on persistence of SARS‐CoV‐2 on food are needed as well as data on whether coronaviruses can be removed from food, e.g. by rinsing with water. However, the faecal–oral route of transmission for COVID‐19 has not been demonstrated and seems unlikely.

### Gastrointestinal tract

In this context, experiments carried out in human intestinal enteroids are of high interest. Mature enterocytes which show a high expression of ACE‐2, the cellular receptor of SARS‐CoV‐2, are the target cells of the virus. SARS‐CoV‐2 enters and leaves the mature enterocytes at the apical membrane facing the gut lumen, which explains the high copy number of viral RNA in the faeces. Viral infectivity is relatively stable in simulated small intestinal juice, but drops in simulated colon juice and is rapidly inactivated in stomach juice (Zang *et al*., 2020). This observation may explain the high faecal viral RNA titre compared to inconsistent infectivity detection reported in faeces by different authors and makes faecal–oral transmission unlikely or at least epidemiologically insignificant. This is in full alignment with the statements of EFSA and other food safety authorities, namely that there is currently no evidence that consumption of food is a likely source or route of transmission of SARS‐CoV‐2 (EFSA, 2020).

## SARS‐CoV‐2 inactivation technologies

### Heat

Heat is an effective means of inactivation for coronaviruses. For MERS‐CoV in cell culture supernatant, 24 and 1 min were necessary to reduce the initial viral titre by 4 log_10_ at 56 and 65°C respectively (Leclercq *et al*., 2014). No infectious MERS‐CoV virus was recovered from dromedary, cow and goat milk spiked with virus and heat‐treated for 30 min at 63°C (van Doremalen *et al*., 2014). Similarly, Rabenau and coauthors reported that for the inactivation of SARS‐CoV in protein‐containing solutions, a treatment at 60°C for at least 30 min is recommended (Rabenau *et al*., 2005). In virus transport medium, SARS‐CoV‐2 was inactivated after 5 min at 70°C (Chin *et al*., 2020). In cell culture supernatant, the virus was inactivated in less than 30 and 15 min at 56°C and 65°C respectively (Wang *et al*., 2020b). This set of data confirm that pasteurization will inactivate SARS‐CoV‐2 and substantiate the WHO statement that SARS‐COV‐2 is not more resistant to heat than the usual bacteria found in food.

### Disinfectants

Human and veterinary coronaviruses are efficiently inactivated by surface disinfection procedures with 62–71% ethanol, 0.5% hydrogen peroxide or 0.1% sodium hypochlorite within 1 min. Other biocidal agents such as 0.05–0.2% benzalkonium chloride are less effective, and 0.02% chlorhexidine digluconate is inefficient (Kampf *et al*., 2020). Efficient inactivation of SARS‐CoV‐2 occurs with WHO‐recommended hand rub formulations and alcohols (Kratzel *et al*., 2020). Both WHO formulations I and II, ethanol and 2‐propanol were shown to inactivate the SARS‐CoV‐2 in 30 s at a minimal final concentration of 30%. No infectious virus was detected after a 5‐min incubation at room temperature in hand soap in two out of three trials (Chin *et al*., 2020). The US Environmental Protection Agency (EPA) has published a list of disinfectant products that meet EPA’s criteria for use against SARS‐CoV‐2 to clean and sanitize food facilities (EPA, 2020). The regular disinfection of high‐contact surfaces is one of the efficient infection control practices to prevent the spread of SARS‐CoV‐2 by fomites. EPA recommends a certain contact time for each compound listed, according to previous inactivation studies done with other viruses. However, for most products, validation data for SARS‐CoV‐2 are not currently available.

### Antimicrobial coatings

Antimicrobial coatings applied to high‐touch surfaces such as door handles, buttons or touch screens may have the potential to reduce SARS‐CoV‐2 titres. For example, copper alloy surfaces may be capable of suppressing virus transmission from fomite surfaces (Scully, 2020). This is enabled by natural corrosion processes on copper triggered by oxidation in both ‘dry’ or humid air, as well as in an infected droplet or aerosol excretion that have settled on fomite surfaces. This process is intrinsic to copper in many environments and can occur without regular human interventions such as regular cleaning. Additional research is needed to confirm the effectiveness of copper alloy surfaces with respect to SARS‐CoV‐2, but data is available on HCoV 229E which was reduced by up to 4 log_10_ units (Warnes *et al*., 2015). A study evaluated the inactivation effect of SurfaceWise2™ (a quaternary ammonium polymer coating) when applied to stainless steel on HCoV 229E (Ikner *et al*., 2020). The coated surfaces were found to be effective against HCoV 229E, reducing the concentration of this virus by 1.3 and 4 log_10_ units after 10 min and 2 h of contact respectively. Another study tested the virucidal activity of immobilized quaternary ammonium compounds (IQACs) coated onto glass and plastic surfaces against enveloped influenza A (H1N1) virus and non‐enveloped poliovirus Sabin1. The IQACs tested were very efficient against the influenza virus: within 2 min, no infective influenza viruses could be retrieved from the coated surfaces (Tuladhar *et al*., 2012). Other coating products, advocating virucidal activity, are commercially available, although no peer‐reviewed validation studies are available to substantiate the claims.

### UV‐C irradiation

UV‐C is known to be a very effective technology to inactivate viruses on smooth surfaces and in clear liquids provided the target is in proximity to the radiation source. Inactivation doses needed are generally higher in liquid than on surfaces. Although there is no current consensus on the amount of UV‐C irradiation required to inactivate SARS‐CoV‐2, a very low UV‐C dose of 1.32–3.20 mJ cm^−2^ was required to inactivate 1 log_10_ (90%) of MS2 Coliphage particles on gel media (Tseng and Li, 2007). On surfaces, additional parameters such as surface structure (smooth *versus* porous), relative humidity of the ambient air and temperature may influence the dose needed for inactivation and exposing a room to UV‐C light will not ensure that all surfaces are reached, due to shadowing effects. Most published studies have tried to determine dosages needed to decontaminate soiled filtering facepiece respirator masks for re‐use in a clinical setting. These types of mask are very porous and correspond to a very difficult surface to treat. For example, for a 3 log_10_ reduction of MS2 Coliphage placed on soiled filtering facepiece respirator (FFR) masks, the necessary UV‐C dose was 4.32 J cm^−2^ (Vo). Comparably, for a variety of mask models, it was found that a 1000 mJ cm^−2^ UV‐C dose conferred a range of 1.42 to 4.84 log_10_ reduction of H1N1 influenza viral load (Mills *et al*., 2018). More data are summarized in a recently released CDC report (CDC, 2020). A preprint report looking at UV‐C dosages to be applied for inactivation of SARS‐CoV‐2 to face shields (smooth plastic surface, easy to treat) and FFR mask (porous, complex surface, difficult to treat) estimated that the safe target UV‐C dosages should be 60 mJ cm^−2^ and 1000 mJ cm^−2^ respectively (Card *et al*., 2020). Compared to surfaces, data on virus inactivation by UV‐C in aerosols are very limited. A study showed that UV‐C irradiation is able to inactivate coronaviruses in aerosols using a murine hepatitis coronavirus as model (Walker and Ko, 2007). Effectiveness was significantly higher than for other virus types (e.g. respiratory adenovirus). According to these results, air sanitation using an irradiation dose of 0.6 mJ cm^−2^ at 254 nm UV‐C may in certain circumstances be an effective tool for inactivating virus in respiratory aerosols, bearing in mind that transmission of SARS‐CoV‐2 by aerosols has not been proven.

### Bipolar ionization

Bipolar ionization is a technology developed for purification of room air. The ionizer generates negative ions, rendering airborne particles/aerosol droplets negatively charged and electrostatically attracts them to a positively charged collector plate. This process involves also the generation of an air current transporting particles through the ionizing device (Wang, 2001). Ionization has been found effective to reduce airborne transmission of influenza A virus in poultry farms (Hagbom *et al*., 2015). Another study investigated the inactivation of MS2 Coliphage through air ions generated by electrical discharge. Bipolar and unipolar treatment reduced MS2 Coliphage by < 2 log_10_ and < 1 log_10_ respectively (Hyun *et al*., 2017). Therefore, currently there is little evidence that ionization can reduce the probability of transmission of SARS‐CoV‐2 via circulating aerosols in rooms. Further studies taking into consideration the impact of air flow, sedimentation kinetics and the inactivation of viruses by the ionization are required. It cannot be excluded that elevated levels of ozone (O_3_) are generated during ionization of air which may represent a health hazard. In addition, it can be expected that ionization generates air flow potentially transporting virus carrying droplets from a contagious person to other subjects in the same room. According to the current state of knowledge, application of this technology for inactivation of SARS‐CoV‐2 in air is premature and further investigations are required.

## Transmission risks for food workers, staff and patrons from COVID‐19

### Slaughterhouses

Crowded and cooled conditions for workers in meat processing facilities make these workplaces high risk for SARS‐CoV‐2 transmission. Slaughterhouses in many countries have reported COVID‐19 cases in the workforce, and qualitative data from the situation in the United States were gathered by CDC during on‐site and remote assessments (Dyal *et al*., 2020). From 9‒27 April 2020, aggregate data on COVID‐19 cases among 115 meat or poultry processing facilities in 19 states were reported to CDC. Among these facilities, COVID‐19 was diagnosed in 4913 (approximately 3%) workers, and 20 COVID‐19‐related deaths were reported. Risk factors included prolonged closeness to other workers for long shifts of up to 12 h, exposure to potentially contaminated shared surfaces such as break room tables or tools, and close contact during transportation in shared vans (Stephenson, 2020). In Germany, > 1300 cases were reported in Nordrhein‐Westfalen and prompted the German Federal Institute for Risk Assessment (BfR) to publish an opinion reiterating that despite the cases seen among workers in German slaughterhouses, meat is not considered a risk for the consumer (Bfr, 2020).

### Living conditions outside of the work environment

Many migrant farm workers live in crowded conditions – such as repurposed shipping containers or shacks in communal housing camps – where physical distancing and adhering to proper sanitary regimes are nearly impossible (Neef, 2020). Most workers share large, dormitory‐style rooms with six to eight workers meeting the minimum cubic volume of space required per worker. The chances for a virus to spread in such conditions are very high. An outbreak exposing workers in a greenhouse in British Columbia, Canada, resulting in 43 positive cases among migrant workers, has demonstrated how susceptible migrant farm workers are in these circumstances (Ella *et al*., 2020). In Singapore, where the COVID‐19 epidemic was first well managed, cases surged thereafter with outbreaks noted in foreign worker dormitories (Chew *et al*., 2020). A marked escalation in the daily number of new COVID‐19 cases was seen in early April 2020. The majority of cases occurred among an estimated 295 000 low‐skilled migrant workers living in foreign worker dormitories. As of 6 May 2020, there were 17 758 confirmed COVID‐19 cases among dormitory workers (88% of 20 198 nationally confirmed cases; Koh, 2020).

### Retail

Retail workers experience potential SARS‐CoV‐2 exposure risk due to the nature of their job and SARS‐CoV‐2 cluster infections in supermarket settings have been reported (Wang *et al*., 2020a). In a report from the United States, among 104 workers tested, 20% tested positive for SARS‐CoV‐2 by RT‐PCR. From the positive cases, 76% were asymptomatic. Employees with direct costumer exposure were five times more likely to test positive for SARS‐CoV‐2 (Lan *et al*., 2020).

### Restaurants and canteens

A data set from a study correlating weekly transmission rate of COVID‐19 in 26 countries for five consecutive weeks by using a machine learning approach with mobility apps from Google and Apple shows that changes of mobility in public places such as restaurants, cafés and theatres were the most important determinant for disease transmission rates, followed by mobility associated with shopping, and then public transport, while workplace attendance showed only a much weaker association with transmission rate changes (Delen *et al*., 2020).The UK Flu Watch group published a study in which 626 participants reported 1005 acute respiratory infections. The researchers asked the participants for activities the week before baseline and the week before an acute infection. Eating out at a restaurant, café or canteen was significantly associated with infection. Restaurant eating risk had a higher odds ratio than travelling on a bus, but a lower one than attending a place of worship. These data were collected for ‘common’ respiratory infections. However, they also seem to fit a pattern observed in COVID‐19 epidemiology (Hayward *et al*., 2020). For example, an outbreak of COVID‐19 was linked to an air‐conditioned restaurant in Guangzhou, China, involving three family clusters who ate at three neighbouring tables (Lu *et al*., 2020).

## Protecting food workers, staff and patrons from COVID‐19

### Fitness to work procedures

Keeping workers in food production and food supply chains healthy and safe is critical to ensure food security during the ongoing pandemic. The WHO issued a guidance document for food businesses outlining measures to protect food workers from contracting COVID‐19 (WHO, 2020a). It is imperative to reinforce personal hygiene measures, awareness of COVID‐19 symptoms and fitness to work procedures, ensuring that infected workers are excluded from food premises. Especially, medical leave policies need to be encouraged and educational materials need to be provided in languages spoken by workers in these settings (Dyal *et al*., 2020). Together, this will help preserve the function of food production facilities which is critical for the entire food supply chain. More general prevention and protection measures applicable to many workplace environments, such as organizational, environmental, and personal measures and adjustments, including the self‐reporting of symptoms, temperature monitoring, enhanced personal hygiene (especially hand washing) and use of PPEs (personal protective equipments), have been comprehensively reviewed (Barnes and Sax, 2020; Cirrincione *et al*., 2020; Rizou *et al*., 2020).

### Physical distancing

Lockdowns in many countries have clearly demonstrated their efficiency to slow the spread of COVID‐19 and push the basic reproduction number R below 1 (where each infected person infects less than one secondary person), and modellers have shown that the relative most efficient individual measure is physical distancing (Davies *et al*., 2020). A meta‐analysis of 172 observational studies investigates the effect of physical distancing, face masks and eye protection use on person‐to‐person transmission of infections using data from SARS, MERS and COVID‐19 studies. Transmission of viruses was lower with physical distancing of > 1 m or more, compared with a distance of < 1 m. The absolute infection risk was 12.8% with shorter distance versus 2.6% with further distance. The analysis showed that further risk reduction could be achieved by extending the distance to 2 m. Every additional 1 m of separation more than doubled the relative protection. The distance effect on infection was stronger for COVID‐19 than for SARS (Chu *et al*., 2020). Practical measures to increase physical distancing between food workers include organizing staff into groups working in different shifts, spacing out workstations and reducing speed of production lines if necessary and avoiding food workers facing each other along the production line (WHO, 2020a). Physical distancing and adhering to proper sanitary regimes are not only important in the work environment, but also need to be ensured outside of work. At retail, maintaining physical distancing and protecting workers by introducing plexiglass barriers at counters and regulating the number of customers who enter a retail store is critical to protect both workers and customers (Desai and Aronoff, 2020; WHO, 2020a).

### Face masks

A study showed that SARS‐CoV‐2 was not transmitted to exposed healthcare workers wearing surgical masks during aerosol‐generating procedures (Rubens *et al*., 2020). Thus, the use of face masks in certain work environments appears to be highly efficient as a means of source control to reduce the spread of the infection in the workforce, especially by minimizing the excretion of respiratory droplets from infected individuals who have not yet developed symptoms or who remain asymptomatic (Sethuraman *et al*., 2020; Treibel *et al*., 2020). Viral shedding in droplets and in aerosol was determined in exhaled breath and coughs from children and adults suffering from common cold. The subjects were naturally infected with seasonal coronavirus, influenza virus and rhinovirus. Wearing a surgical face mask reduced coronavirus titre determined by RT‐PCR both in droplets and in aerosols (Leung *et al*., 2020). It is not known how much the use of masks can contribute to a decrease in transmission in addition to the other countermeasures. The use of face masks should be considered as a complementary measure and not as a replacement for established preventive measures, such as physical distancing, respiratory etiquette, meticulous hand hygiene and avoiding touching the face, nose, eyes and mouth. Appropriate use of face masks is key for the effectiveness of the measure and can be improved through education campaigns (ECDC, 2020). From epidemiological data, countries that have been most effective in reducing the spread of COVID‐19 have implemented universal masking, including Taiwan, Hong Kong, Singapore and South Korea. Taiwan despite its closeness to China has achieved control without lockdown, adding economic arguments to the universal masking strategy (Prather *et al*., 2020).

### Ventilation in closed settings

Quantitative Microbial Risk Assessment developed for MERS (Adhikari *et al*., 2019) showed that sick people excreting virus by coughing, cause short‐ and long‐range airborne exposures of people sharing the same room. The authors recommended a minimum room ventilation rate of six air changes per hour to minimize recirculation of pathogen‐bearing droplets and meticulous environmental cleaning for preventing transmission in healthcare settings (Oh *et al*., 2018). In the current pandemic, ventilation practices should be reviewed, and ventilation maximized (Morawska and Cao, 2020), especially in closed and crowded settings such as canteens and restaurants. Tracer gas measurements and computational fluid dynamics (CFD) simulations were used to predict the spread of fine droplets exhaled by the index patient and the detailed airflow pattern at the restaurant outbreak in Guangzhou (Li *et al*., 2020b). The authors observed that high concentration of the simulated contamination resulted from lack of outdoor supply. Indeed, the measured average air flows around 1 l s^−1^ were considerably lower than the 8–10 l s^−1^ per person required by most authorities or professional societies (American Society of heating etc). The results of the study do not show that long‐range aerosol transmission of SARS‐CoV‐2 can occur in any indoor space, but that transmission may occur in a crowded and poorly ventilated space. Indeed, when the ventilation rate of the room is too low, viral concentrations within the room may raise to levels similar than in exhaled air from a patient. Hence, in theory, even if an infectious agent is not typically (i.e. under adequate ventilation) transmitted by a long‐range aerosol mechanism, the spatial extent of transmission increases if the ventilation rate is very low. The authors refer to such transmission as an extended short‐range aerosol transmission mechanism.

## Collateral damage from the COVID‐19 pandemic

### Disruption of food supply chains and food security

The COVID‐19 epidemic has many ramifications for our society. A joint statement from the FAO, the WHO and the WTO was posted on March 30 emphasizing that millions of people around the world depend on international trade for their food security and livelihoods (FAO, 2020). As countries enact measures against the COVID‐19 pandemic, governments should minimize impact on food supply and unintended consequences on global trade and food security (HLPE, 2020). Indeed, the chief economist of FAO has warned of isolationism as a response to the COVID‐19 pandemic. Food production is globally connected, so interrupting supply chains will threaten food security worldwide. The prospects for cereal, rice and soya harvests are good, but low oil price and panic buying has increased the price of wheat and rice. Food is decaying on farms, on markets and in ports. Policy makers should declare that dock and farm workers, including migrant workers, are essential personnel for the survival of the population, similarly as health personnel. Currently, food loss is estimated at $400 billion annually, which could feed 1.2 billion people a year. If this wasting of food increases with isolationism, a food crisis could follow the pandemic (Torero, 2020). In the United States, the USDA programme that usually provides school breakfasts and lunches to 35 million children has been interrupted during school closures which affects the nutrition of children from low‐income families. Public health specialist discusses ‘Grab‐n‐Go’ meal sites at school bus stops to prevent food shortage in poor families (Dunn *et al*., 2020). This problem is accentuated in countries that are unable to satisfy the food needs of their population in normal times.

### Quality assurance

FDA announced that during the COVID‐19 pandemic, inspectors would focus their inspections on critical missions and postpone routine surveillance inspections (FDA, 2020a). Similarly, the European Commission has given member states more flexibility to do official controls in the food supply chain during the ongoing epidemic (EC, 2020). Restrictions are also impacting on the world of third‐party certification, including food businesses, certification bodies, accreditation bodies and training organizations. A COVID‐19 position paper for FSSC 22000 licensed training organizations has been published to address issues such as validity extension of existing FSSC 22000 certificates (FSSC, 2020). Hence, vigilance of food manufacturers must remain high and strict adherence to quality assurance and quality control procedures must be enforced despite the pandemic to ensure that products put on the market are compliant and safe. Food safety authorities, organizations and academic institutions such as WHO, FDA and Cornell University College of Agriculture in the United States have helped the food sector to adapt to this new situation and put in place additional control measures by issuing guidance documents and hosting webinars including Q&A sessions for food companies (MatrixSciences, 2020; WHO, 2020a; FDA, 2020b).

### Misperceptions about COVID‐19 and food safety risks

New research by University College London (UCL) has found people hold potentially harmful misperceptions about COVID‐19 and food safety/nutrition (UCL, 2020). The researchers designed a survey that contained 25 statements with participants answering whether they viewed the statements as ‘correct’, ‘incorrect’ or ‘not sure’. Analysing answers by 3781 respondents, the team found 43% of participants wrongly believe that it is necessary to wash fruits and vegetables with soap or diluted bleach to supposedly remove potential COVID‐19 viral particles from food. This resonates with a report from the United States which showed that the daily number of calls to poison centres increased sharply at the beginning of March 2020 for exposures to both cleaners and disinfectants (Chang *et al*., 2020). Similar to the UCL survey, a recent US internet panel survey identified gaps in knowledge about safe preparation, use, and storage of cleaners and disinfectants (Gharpure *et al*., 2020). Approximately one‐third of survey respondents engaged in non‐recommended high‐risk practices with the intent of preventing SARS‐CoV‐2 transmission, including using bleach on food products. Public messaging should continue to emphasize that there is currently no evidence that food is a likely source or route of transmission of the virus (EFSA, 2020; Li *et al*., 2020a) and that evidence‐based, safe cleaning and disinfection practices to prevent SARS‐CoV‐2 transmission in households include hand hygiene and cleaning and disinfection of high‐touch surfaces. WHO recommends to wash fruit and vegetables with potable water (WHO, 2020a). Indeed, rinsing strawberries with water was shown to remove > 1 log_10_ MS2 Coliphage and enteric viruses such as hepatitis A virus from the food surface (Zhou *et al*., 2017). Additionally, WHO recommends to follow the 5‐Keys to Safer Food (keep clean; separate raw and cooked; cook thoroughly; keep food at safe temperatures; and use safe water and raw materials) (WHO, 2019).

## Conclusion

The amount of scientific data published since the appearance of SARS‐CoV‐2 in China late in 2019 has grown exponentially (Stoye, 2020). Scientists and medical doctors have shared information globally at a speed not seen in the past. New data are available in preprint format ahead of peer review and acceptance by a scientific journal which raises questions about low quality data being circulated. However, this new way of working has proven extremely powerful in the current pandemic to share experiences, new knowledge, practices and control measures as quickly as possible. Many questions around the persistence and inactivation of SARS‐CoV‐2 have already been answered such as inactivation by commonly used disinfectants and hand sanitizers. Others will require further research to fill current knowledge gaps, especially determining the minimal infectious dose for humans and resolving uncertainties around the role of additional modes of transmission. Especially, the transfer rate of virus from surface to hand to face needs to be established for various types of surfaces and foods to assess the risk and establish the importance of fomite transmission in the ongoing pandemic. Better risk assessments will also help to determine which impact the usage of alternative techniques such as air sanitation devices may have in mitigating the risk of SARS‐CoV‐2 transmission in food production areas and at retail. Actors along the food chain have generally adapted quickly and adequately to the pandemic, and food production has not been severely disrupted so far. However, the pandemic is not over and all actors along the food supply chain need to remain very vigilant, especially in controlling outbreaks in the workforce (e.g. in slaughterhouses), to avoid collateral damage such as supply chain shortages or break‐down, appearance of fraudulent ingredients and issues with quality and safety of raw materials or finished products. Additionally, researchers from Johns Hopkins University predict between 250 000 and 1 mio child deaths not from COVID‐19 directly, but from reduced antibiotic coverage, lack of oral rehydration treatment, interrupted vaccination programmes and increased childhood malnutrition (Roberton *et al*., 2020). This global threat will need to be tackled globally and political leaders have a big role to play, including counter disinformation, to avoid a food crisis in the near future (Ball and Maxmen, 2020).

## Conflict of interest

Dr. Sophie Zuber is employee of the Nestlé Research Center in Lausanne/Switzerland; Dr. Harald Brüssow is a former employee of the Nestlé Research Center in Lausanne/Switzerland and currently paid as consultant of Nestlé on the scientific literature about the COVID‐19 pandemic.
